# Global trends and performances of infrared imaging technology studies on acupuncture: a bibliometric analysis

**DOI:** 10.3389/fnins.2024.1387752

**Published:** 2024-04-19

**Authors:** Yuanyuan Feng, Yunfan Xia, Binke Fan, Shimin Li, Zuyong Zhang, Jianqiao Fang

**Affiliations:** ^1^The Third Clinical Medical College of Zhejiang Chinese Medical University, Hangzhou, China; ^2^The Third People’s Hospital of Hangzhou, Hangzhou, China; ^3^Key Laboratory of Acupuncture and Neurology of Zhejiang Province, Department of Neurobiology and Acupuncture Research, The Third Clinical Medical College, Zhejiang Chinese Medical University, Hangzhou, China

**Keywords:** bibliometric analysis, acupuncture, IRT, infrared imaging technology, near infrared spectroscopy, fNIRS, manual needle acupuncture, temperature

## Abstract

**Objectives:**

To summarize development processes and research hotspots of infrared imaging technology research on acupuncture and to provide new insights for researchers in future studies.

**Methods:**

Publications regarding infrared imaging technology in acupuncture from 2008 to 2023 were downloaded from the Web of Science Core Collection (WoSCC). VOSviewer 1.6.19, CiteSpace 6.2.R4, Scimago Graphica, and Microsoft Excel software were used for bibliometric analyses. The main analyses include collaboration analyses between countries, institutions, authors, and journals, as well as analyses on keywords and references.

**Results:**

A total of 346 publications were retrieved from 2008 to 2023. The quantity of yearly publications increased steadily, with some fluctuations over the past 15 years. “Evidence-Based Complementary and Alternative Medicine” and “American Journal of Chinese Medicine” were the top-cited journals in frequency and centrality. China has the largest number of publications, with the Shanghai University of Traditional Chinese Medicine being the most prolific institution. Among authors, Litscher Gerhard from Austria (currently Swiss University of Traditional Chinese Medicine, Switzerland) in Europe, was the most published and most cited author. The article published by Rojas RF was the most discussed among the cited references. Common keywords included “Acupuncture,” “Near infrared spectroscopy,” and “Temperature,” among others. Explore the relationship between acupoints and temperature through infrared thermography technology (IRT), evaluate pain objectively by functional near-infrared spectroscopy (fNIRS), and explore acupuncture for functional connectivity between brain regions were the hotspots and frontier trends in this field.

**Conclusion:**

This study is the first to use bibliometric methods to explore the hotspots and cutting-edge issues in the application of infrared imaging technology in the field of acupuncture. It offers a fresh perspective on infrared imaging technology research on acupuncture and gives scholars useful data to determine the field’s hotspots, present state of affairs, and frontier trends.

## 1 Introduction

Acupuncture is a millennia-old Chinese medical treatment. Acupuncture encompasses a variety of procedures, such as acupuncture, moxibustion, cupping, and bloodletting. It is to stimulate specific body points by different means to derive therapeutic benefits. Due to its undeniable therapeutic benefits in a variety of neurological and pain-related diseases, it is now rapidly gaining popularity in the West as a complementary therapy.

In recent decades, infrared imaging technology has been used more and more in the field of acupuncture (Hillen et al., 2020; Fu et al., 2024). The existing infrared imaging technologies for acupuncture can be mainly divided into two methods: near infrared spectroscopy and dynamic infrared thermography. Infrared thermography technology (IRT) is a non-invasive technology that utilizes naturally emitted infrared radiation from the skin surface to generate real-time digital images. And more and more clinical research has used fNIRS, a non-invasive, motion-insensitive, functional brain imaging technique that uses near-infrared light to monitor hemodynamic changes in the cerebral cortex. However, with the increase in research in this area, little attention was paid to the summary of relevant literature and the estimation of the general situation in the infrared imaging technology field of acupuncture.

Bibliometric methods are quantitative methods that use statistical and mathematical tools to examine vast amounts of literature on a particular topic (Park et al., 2021). By quantifying the co-occurrence of data in the literature, such as authors, organizations, countries, keywords, and number of citations, we can possibly comprehend various network relationships (De Bellis, 2009). It is worth noting that CiteSpace and VOSviewer are two widely used information visualization software. They can intuitively show the research hot spots and development trends in the field they study.

Thus, in this study, knowledge maps were utilized to analyze the current research hotspots and trends of infrared imaging technology research on acupuncture throughout time using CiteSpace, VOSviewer, Scimago Graphica, and Microsoft Excel software.

## 2 Methods

### 2.1 Search strategy

The data was retrieved from the WoSCC via the Zhejiang Chinese Medical University Library website on October 12th, 2023, encompassing Science Citation Index Expanded, Current Chemical Reactions, and Index Chemicus. “Infrared Imaging Technology” and “Acupuncture Therapy” were included in the search strategy, without restrictions on the languages. Duplicate studies were eliminated. Table 1 lists the specific search tactics and outcomes.

**Table 1:** The topic search query.

Set	Results	Search query
#1	25,013	TS = (“Acupuncture Therapy” OR “Acupuncture” OR “Cupping Therapy” OR “Bloodletting” OR “acupuncture” OR “electroacupuncture” OR “moxibustion” OR “moxa” OR “cupping” OR “bloodletting” OR “blood-letting” OR “pricking blood” OR “pyonex” OR “acupressure” OR “meridian” OR “meridians” OR “acupoint” OR “acupoints”) Indexes = SCI-EXPANDED,SSCI,A&HCI,ESCI Timespan =2008–2023
#2	547,740	(((((((((((((TS = (Infrared thermal imag*)) OR TS = (infrared imag*)) OR TS = (therm* imag*)) OR TS = (Infrared thermography)) OR TS = (IRT)) OR TS = (Thermographic mapping)) OR TS = (Thermographic patterns)) OR TS = (Thermal imaging camera)) OR TS = (therm* camera*)) OR TS = (Infrared spectrophotometry)) OR TS = (infrared camera)) OR TS = (Infrared*)) OR TS = (Thermology)) Indexes = SCI-EXPANDED,SSCI,A&HCI,ESCI Timespan = 2008–2023
#3	346	#1 AND #2

### 2.2 Analysis tool

We used VOSviewer 1.6.19, CiteSpace 6.2.R4 to create the knowledge network map, Scimago Graphica and Microsoft Excel software for the statistical analysis and geographical visualization. The parameters of CiteSpace included time slices from 2008 to 2023, factor *k* = 25, and Pathfinder, pruning sliced networks and pruning merged network. Every node in the visualization represented a unit, and its size revealed the frequency. A node’s large size and warm color suggested that it had experienced a recent high-frequency explosion, while the nodes with a purple outer circle often represented the literature with higher centrality. The thicker the lines between nodes, the higher the degree of cooperation or co-occurrence between them.

VOSviewer (Netherlands’ Leiden University) was used to analyze co-authors and cited references. A node represents an author or a reference, while the size of the node reflects its frequency. The link strength between nodes represented the degree of collaboration between authors or cited literature. Scimago Graphica^1^ and VOSviewer were used to analyze national geographic distribution, while Microsoft Excel software was used to visualize the annual publication.

## 3 Results

### 3.1 General information

#### 3.1.1 Annual publications

**Figure 1:**
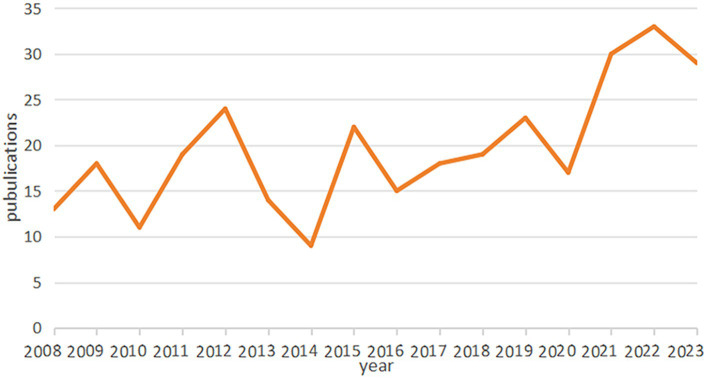
The trend analysis of annual publications engaged in the application of infrared imaging technology in acupuncture from 2008 to 2023.

A total of 346 publications were included in this study. Figure 1 shows that the number of publications fluctuated over the past 15 years, but the overall trend was upward. It was noteworthy that the number of increases in publications surged from 2020, culminating in a peak of 33 articles in 2022. The trend of publications indicated that the application of infrared imaging technology in the field of acupuncture has received more attention recently.

#### 3.1.2 Analysis of country

**Figure 2:**
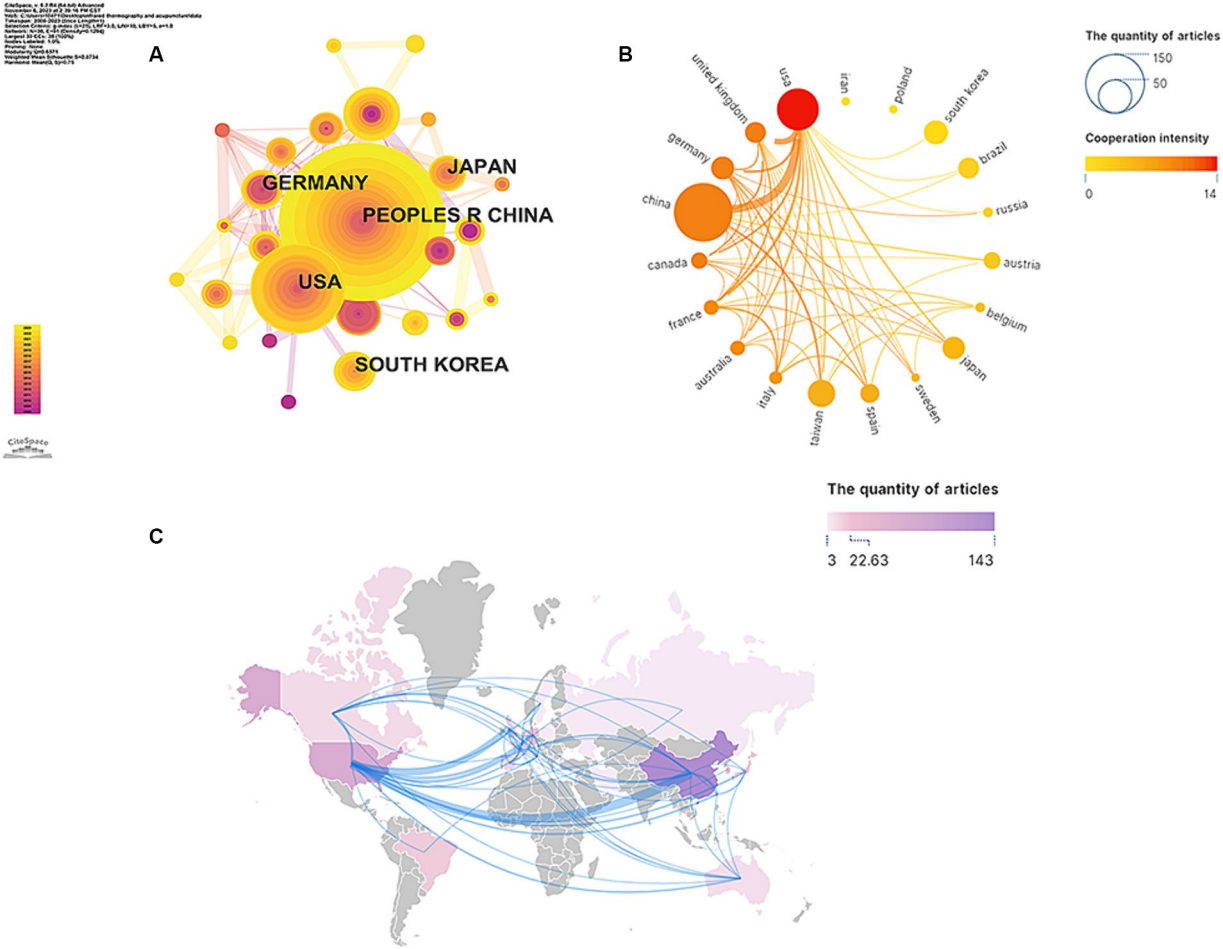
Cooperation between countries/regions **(A,B)**. The cooperation networks between different countries/regions related to the application of infrared imaging technology in acupuncture from 2008 to 2023. **(C)** Distribution of countries/regions.

From 2008 to 2023, a total of 38 countries made significant contributions to this research field (as shown in Figure 2A and Table 2). The top five countries with the highest publication output were China (169), the United States (73), South Korea (24), Germany (22) and Japan (21), while the remaining countries had fewer than 20 publications. In terms of country centrality, the United States (0.36) was in first place, followed by Germany (0.09) and China (0.08). Subsequently, we constructed cooperative network based on the quantity and correlation of publications in every country (Figures 2B,C). It can be observed from Figure 2C that the United States has a close cooperative relationship with China and England.

**Table 2:** Top 5 publications and centrality of countries of infrared imaging technology in acupuncture.

Rank	Publications	Country	Citations	Centrality	Country	Citations
1	169	China	2,265	0.36	United States	2,278
2	73	United States	2,278	0.15	Spain	200
3	24	South Korea	253	0.09	Germany	582
4	22	Germany	582	0.08	China	2,265
5	21	Japan	336	0.08	France	313

#### 3.1.3 Analysis of institution

**Figure 3:**
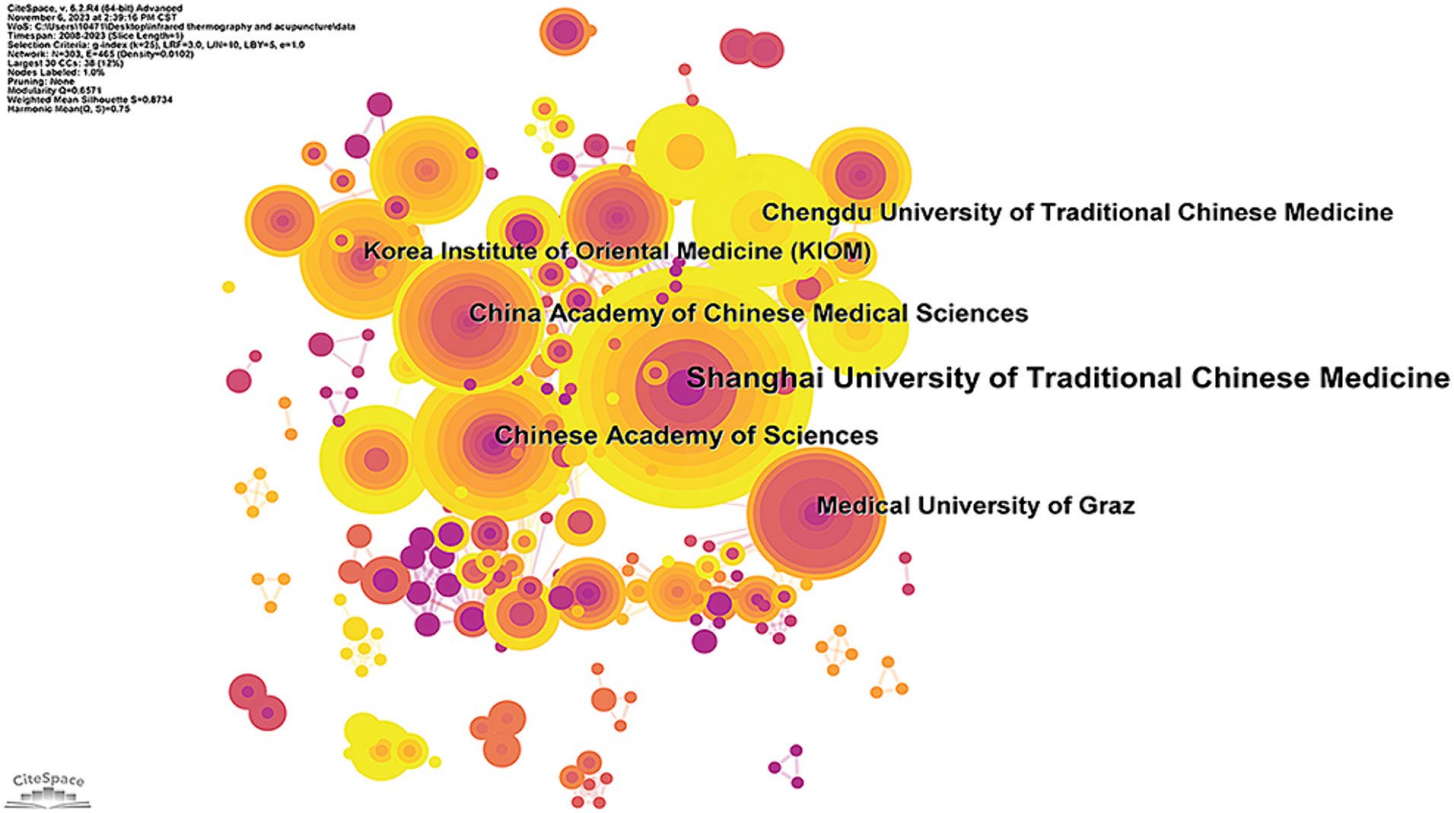
An institution cooperation map related to the application of infrared imaging technology in acupuncture from 2008 to 2023.

As shown in Figure 3 and Table 3, 303 institutions have made contributions in this field. The top three institutions with the most published literatures were *Shanghai University of Traditional Chinese Medicine (20)*, *Chinese Academy of Sciences (13)*, and *China Academy of Chinese Medical Sciences (12)*. Apart from this, the top three institutions with the highest centrality were *Harvard University (0.13)*, *Beijing University of Chinese Medicine (0.11)*, and *Medical University of Graz (0.11)*.

**Table 3:** Top 10 publications and centrality of institutions of infrared imaging technology in acupuncture.

Rank	Publications	Institution	Rank	Centrality	Institution
1	20	Shanghai University of Traditional Chinese Medicine	1	0.13	Harvard University
2	13	Chinese Academy of Sciences	2	0.11	Medical University of Graz
3	12	China Academy of Chinese Medical Sciences	3	0.11	Beijing University of Chinese Medicine
4	11	Chengdu University of Traditional Chinese Medicine	4	0.1	Chinese Academy of Sciences
5	11	Medical University of Graz	5	0.09	China Academy of Chinese Medical Sciences
6	10	Korea Institute of Oriental Medicine (Kiom)	6	0.09	Hong Kong Baptist University
7	9	Beijing University of Chinese Medicine	7	0.08	China Medical University Taiwan
8	9	Institute of Acupuncture & Moxibustion	8	0.07	Institute of acupuncture & moxibustion
9	9	Daejeon University	9	0.7	National Taiwan university
10	8	Harvard University	10	0.7	Johns Hopkins university

#### 3.1.4 Analysis of authors

**Figure 4:**
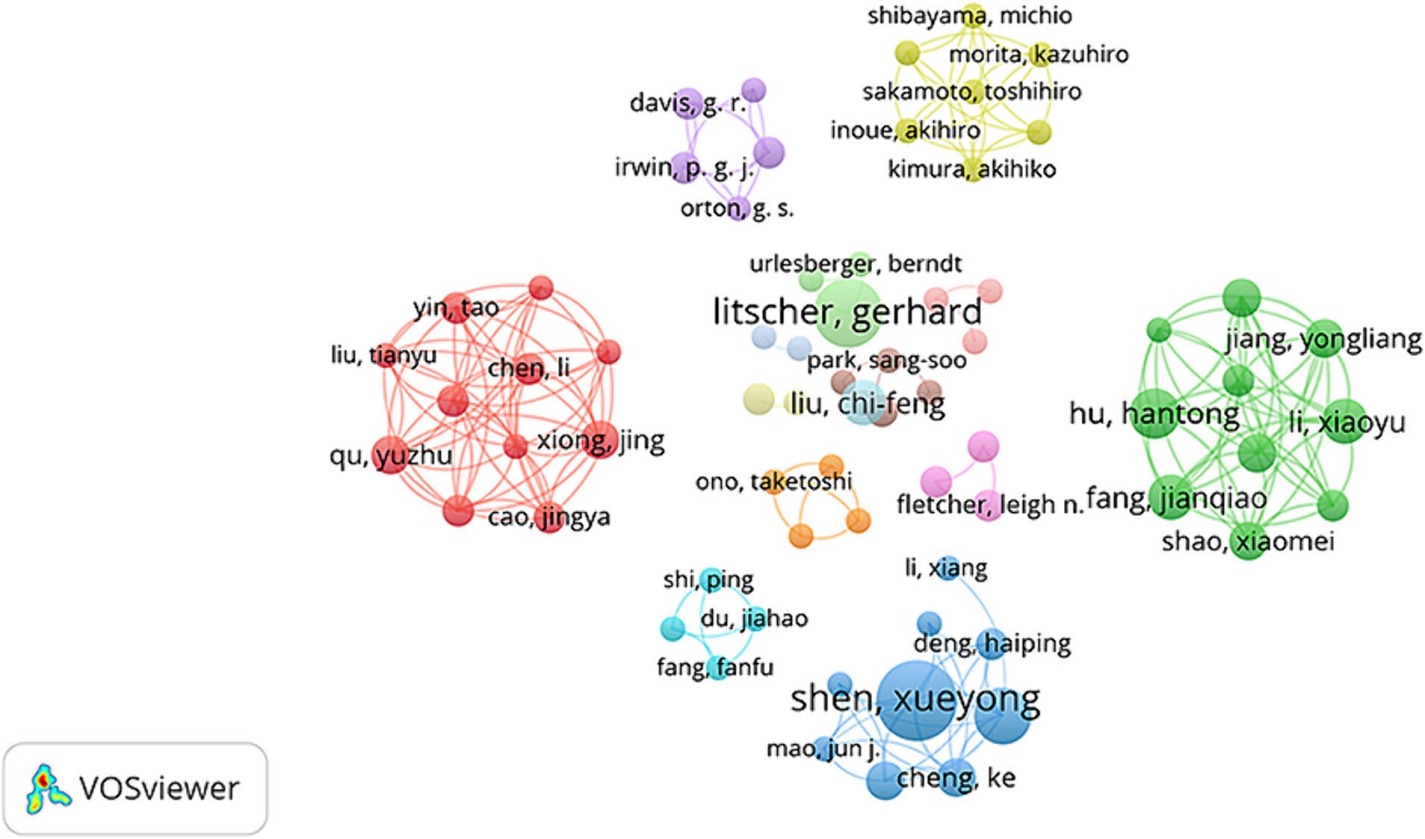
Map of co-authors related to the application of infrared imaging technology in acupuncture from 2008 to 2023.

There were 423 authors who participated in the study of infrared imaging technology research on acupuncture (as shown in Figure 4 and Table 4), among whom 7 authors had published more than five articles. Figure 4 was designed to reveal the highly productive authors and the relationships between authors. The node size represents the number of articles published by the author. Litscher, Gerhard (7), and Shen Xueyong (6) were the top two productive researchers, followed by Liu Chi-Feng (5), Xiong Jing (5), and Cheng Ke (5). The figure illustrates the lack of joint publications among prominent writers who have conducted research in the field of acupuncture infrared imaging technology. As the top two productive authors in the field, Litscher, Gerhard and Shen Xueyong both used IRT to study the temperature distribution of different acupuncture points, trying to prove the existence of acupuncture points and the effectiveness of acupuncture from the infrared signature and temperature (Wang et al., 2012; Huang et al., 2013; Zheng et al., 2013). However, Litscher, Gerhard is more concerned about laser acupuncture. He has used IRT to detect the effect of laser acupuncture on acupoint temperature in several experiments (Litscher et al., 2011; Kurath-Koller et al., 2015; Lan et al., 2019). Shen analyzed different types of moxibustion from the perspective of infrared spectroscopy to explore the mechanism of moxibustion (Deng and Shen, 2013). He found that laser moxibustion has a positive effect on cancer-related fatigue and knee osteoarthritis pain (Mao et al., 2016; Li et al., 2020).

**Table 4:** The top 5 publication of authors and co-cited authors of infrared imaging technology in acupuncture.

Rank	Publications	Author	Citations	Co-cited Authors
1	7	Litscher, Gerhard	29	Litscher G
2	6	Shen, Xueyong	21	Shen XY
3	5	Liu, Chi-Feng	17	Rojas RF
4	5	Xiong, Jing	17	Macpherson H
5	5	Cheng, Ke	11	Zhao L

#### 3.1.5 Analysis of journals and co-cited academic journals

A total of 185 journals had published articles related to the application of infrared imaging technology in acupuncture. Table 5 showed the top 10 academic journals that published articles in the field of acupuncture infrared imaging technology. Evidence-Based Complementary and Alternative Medicine (36) was the most productive journal, followed by Journal of Traditional Chinese Medicine (10), Acupuncture in Medicine (7), Lasers in Medical Science (7) and Acupuncture & Electro-Therapeutics Research (6).

**Table 5:** Top 10 journals of infrared imaging technology in acupuncture.

Rank	Publications	Journal	IF	Country
1	36	Evidence-Based Complementary and Alternative Medicine	2.65	England
2	10	Journal of Traditional Chinese Medicine	2.547	China
3	7	Acupuncture in Medicine	2.5	England
4	7	Lasers in Medical Science	2.1	England
5	6	Acupuncture & Electro-Therapeutics Research	0.3	United States
6	5	American Journal of Chinese Medicine	5.7	United States
7	5	Frontiers in Neuroscience	4.3	Switzerland
8	5	Medicine	1.6	United States
9	4	Journal of Pain Research	2.7	England
10	4	Plos One	3.7	United States

**Figure 5:**
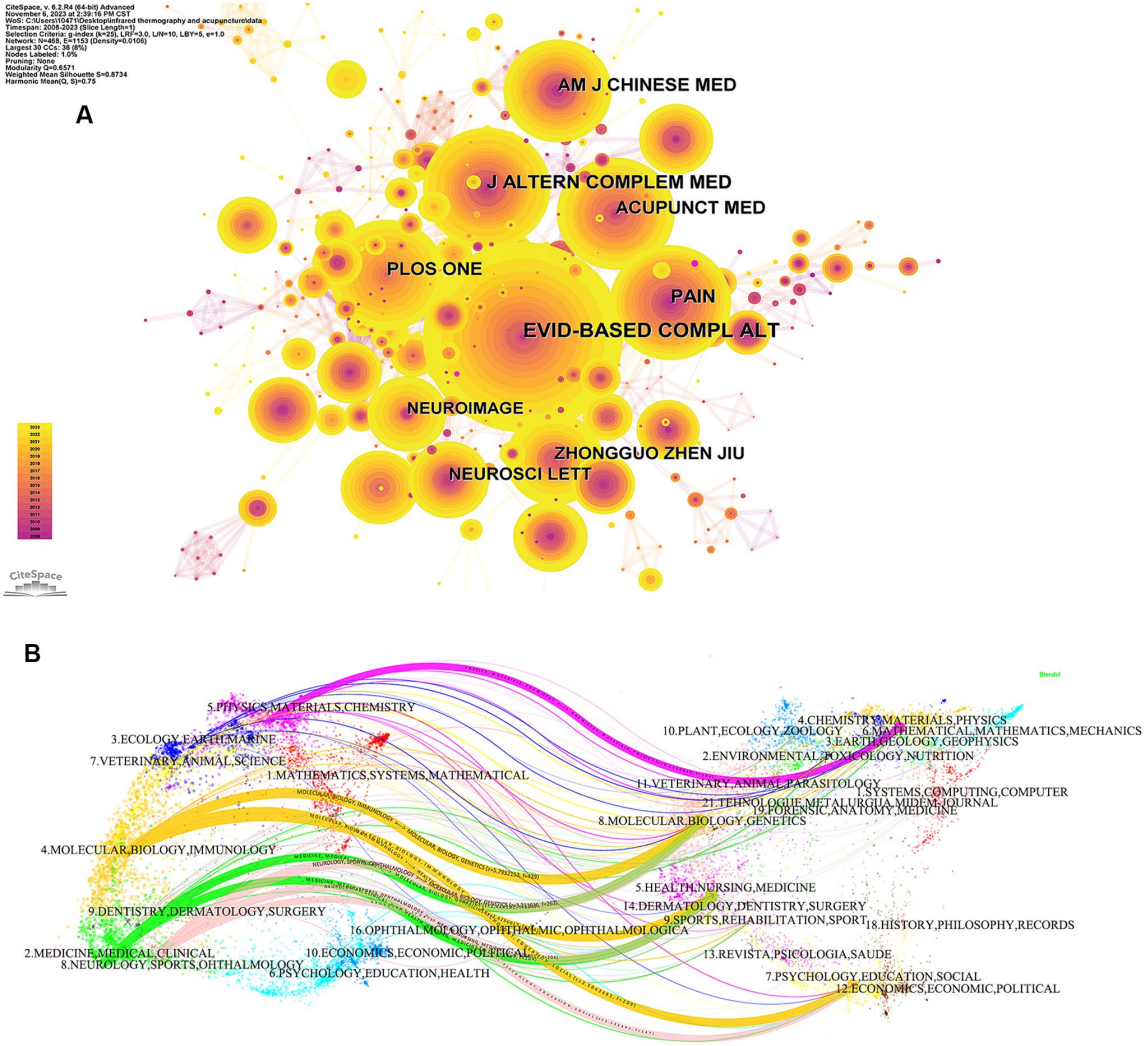
**(A)** Cited journal maps related to the application of infrared imaging technology in acupuncture from 2008 to 2023. **(B)** A dual-map overlay of journals.

The cited journal map is displayed in Figure 5A. Table 6 shows the top 5 cited journals of infrared imaging technology in acupuncture. It is obviously true that Evidence-Based Complementary and Alternative Medicine (124) and the American Journal of Chinese Medicine (0.17) were the top journals in terms of frequency and centrality, respectively. These results indicated that the articles in the field of acupuncture infrared imaging technology in these two journals are highly persuasive and representative.

**Table 6:** Cited journals and centrality of infrared imaging technology in acupuncture.

Rank	Frequency	Journal	IF	Country	Rank	Centrality	Journal	IF	Country
1	124	Evidence-Based Complementary and Alternative Medicine	2.65	England	1	0.17	American Journal of Chinese Medicine	5.7	United States
2	79	Journal of Alternative and Complementary Medicine	2.6	United States	2	0.15	Plos one	3.7	United States
3	75	Pain	7.4	Netherlands	3	0.14	Nature	64.8	England
4	73	Acupunct Med	2.5	England	4	0.13	Lancet	168.9	England
5	72	Plos One	3.7	United States	5	0.12	Pain	7.4	Netherlands

Using CiteSpace, a dual-map overlay with the citation and cited journals was created (Figure 5B). The left side represents the citing journals and the right side the cited journals. As shown in the map, the green paths illustrate that studies published in “medicine, medical, clinical” journals frequently reference research published in “Molecular, Biology, Genetics” and “Health, Nursing, Medicine.” Orange paths indicate studies published in “Molecular, Biology, Genetics,” “Health, Nursing, Medicine” and “Psychology, Education, social” journals that are cited for research in “Molecular, Biology, Immunology” journals.

### 3.2 Research hotspots and global trend

#### 3.2.1 Analysis of co-cited authors

The co-cited authors were presented in Table 4. In terms of the number of publications, Litscher G (29) ranked first among the cited authors, followed by Shen XY (21), and Rojas RF (17). Among them, Litscher G has recently focused on laser acupuncture with thermal imaging. And Shen XY was united in his commitment to laser moxibustion. Rojas RF, from the University of Canberra, was dedicated to using fNIRS to study acupuncture analgesia and its relationship with prefrontal cortex (PFC).

#### 3.2.2 Analysis of co-occurring keywords

**Figure 6:**
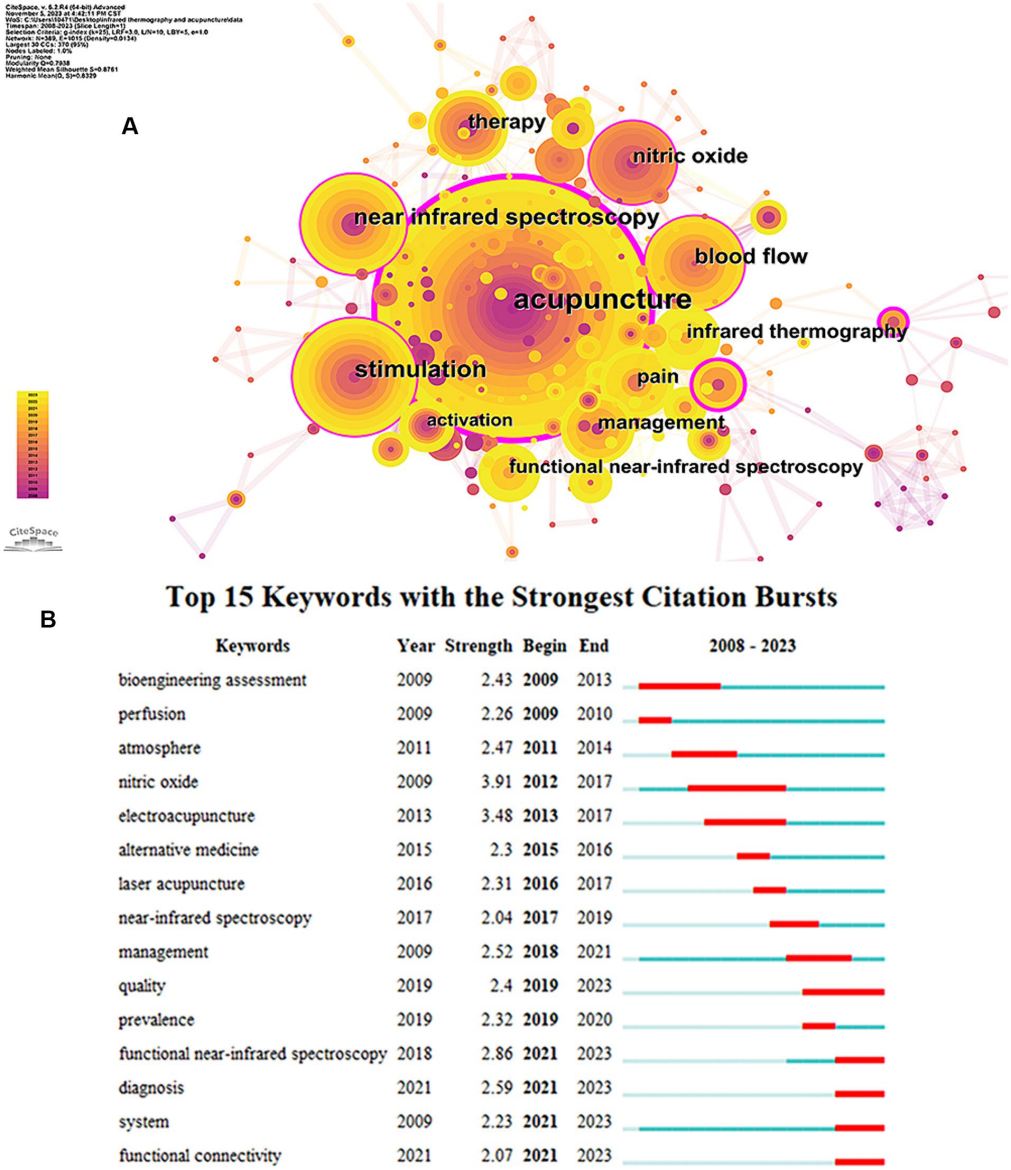
**(A)** Map of keywords related to the application of infrared imaging technology in acupuncture from 2008 to 2023. **(B)** Top 15 keywords with the strongest citation bursts. The red color represents the keyword was cited in high frequency and the blue color represents in low frequency.

Research hotspots and trends in specific fields are usually obtained by analyzing keywords with high centrality and frequency. Figure 6A shows the keyword co-occurrence map. Table 7 lists the top 10 keywords for frequency and centrality. Over the past 15 years, the most frequently occurring keywords in the field of acupuncture infrared imaging technology were “Acupuncture,” “Stimulation,” “Near infrared spectroscopy,” “Blood flow,” and “Nitric oxide.” The five with the highest centrality keywords were “Acupuncture,” “Temperature,” “Model,” “Stimulation” and “Nitric oxide.”

**Table 7:** Top 10 keywords of infrared imaging technology in acupuncture.

Rank	Frequency	Keywords	Centrality	Keywords
1	44	Acupuncture	0.41	Acupuncture
2	20	Stimulation	0.27	Temperature
3	17	Near infrared spectroscopy	0.22	Model
4	16	Blood flow	0.16	Stimulation
5	14	Nitric oxide	0.14	Nitric oxide
6	13	Therapy	0.13	Near infrared spectroscopy
7	12	Pain	0.13	Blood flow
8	12	Management	0.09	Management
9	11	Infrared thermography	0.07	Therapy
10	10	Functional near-infrared spectroscopy	0.07	Pain

#### 3.2.3 Analysis of co-cited references

Two (or more) papers cited by one or more papers at the same time is called reference co-citation and is used to describe the degree of correlation between the papers. Co-citation implies a strong correlation between the cited literature and the corresponding research, indicating that the literature typically encompasses high-quality content with substantial impact within a specific research domain.

**Figure 7:**
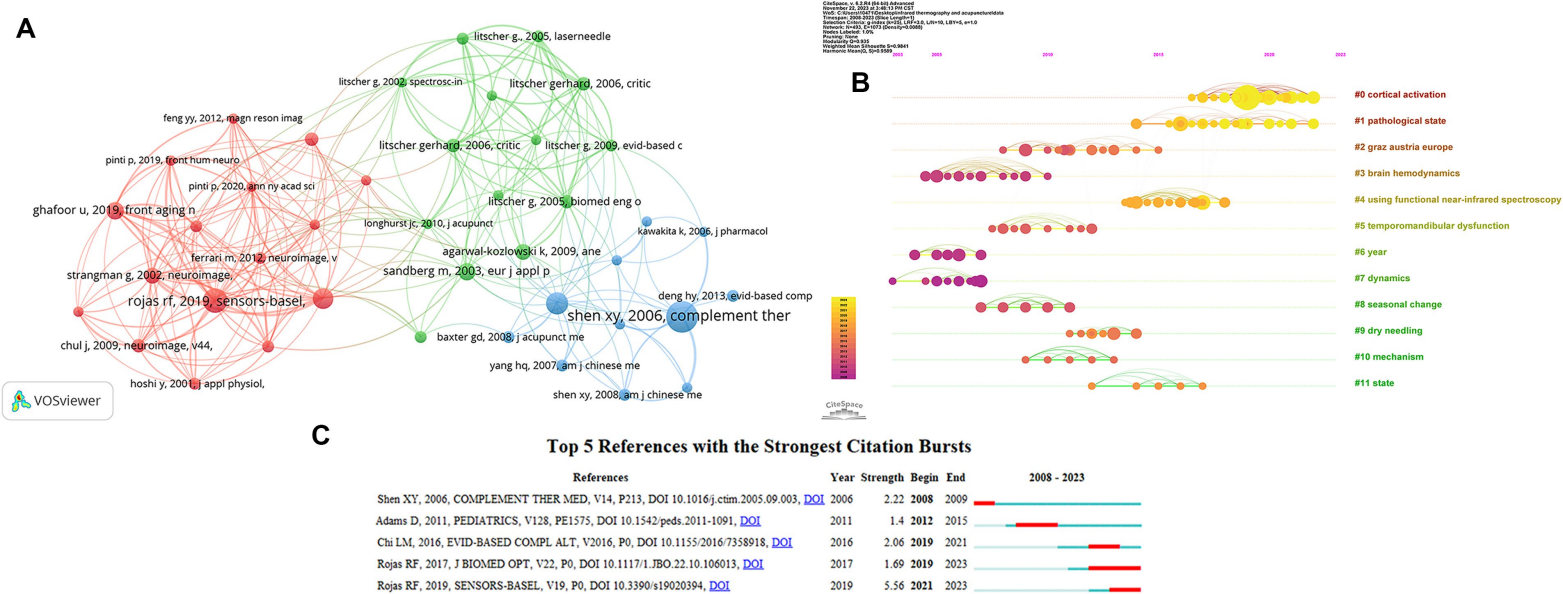
**(A)** Map of cited references related to the application of infrared imaging technology in acupuncture from 2008 to 2023. **(B)** Timeline view of infrared imaging technology in acupuncture. **(C)** Top 5 references with strong citation burstiness. The red bars mean some references cited frequently; the blue bars were references cited infrequently.

Table 8 and Figure 7A presented the top five cited references. The study published by Raul Fernandez Rojas in 2019 had the highest co-citation counts, followed by Ghafoor U, (2019) and Shen XY, (2006). Fernandez Rojas et al. (2019) conducted research that identified the anesthetic effect of the Hegu point based on the hemodynamic response as measured by fNIRS. The study published by Ghafoor U and his team in 2019 observed through fNIRS that after acupuncture treatment, the mean hemodynamic response of working memory tasks and functional connectivity in the prefrontal cortex of patients with mild cognitive impairment increased (Ghafoor et al., 2019). Shen XY and his team published in 2006 revealed that thermal action of the traditional moxa stick was more potent than that of indirect moxibustion by using a highly sensitive, infrared-spectrum detection device (Shen et al., 2006).

**Table 8:** The top 5 citation of infrared imaging technology in acupuncture.

Rank	Co-citation counts	Cited reference	Author and publication year
1	13	Cortical network response to acupuncture and the effect of the hegu point: an fNIRS study	Rojas RF, (2019)
2	9	Effects of acupuncture therapy on MCI patients using functional near-infrared spectroscopy	Ghafoor U, (2019)
3	4	An infrared radiation study of the biophysical characteristics of traditional moxibustion	Shen XY, (2006)
4	4	Toward a functional near-infrared spectroscopy-based monitoring of pain assessment for nonverbal patients	Rojas RF, (2017)
5	4	The effectiveness of cupping therapy on relieving chronic neck and shoulder pain: a randomized controlled trial	Chi LM, (2016)

The fourth article was published in 2017 by Raul Fernandez Rojas, which indicated that potential applications of fNIRS in the development of a physiologically based diagnosis of human pain that would benefit vulnerable patients who cannot self-report pain (Fernandez Rojas et al., 2017). The last article was a randomized controlled trial published in 2016 by Lee-Mei Chi, which identified the effectiveness of cupping therapy (CT) in changes in skin surface temperature (SST) for relieving chronic neck and shoulder pain (NSP) among community residents (8).

Citation burst refers to citation being cited a lot in a short period of time. Through the citation burst, we can focus on the research hotspots of this period and judge the future development trend of this field. Figure 7C presents the top 5 references with strong citation burstiness, four of which had the same results as the highly co-cited references. Two articles published by Raul Fernandez Rojas in 2017 and 2019 received widespread attention starting in 2019 and 2021, respectively, and continue to this day.

To investigate the clusters of a cited-references, the log-likelihood test (LLR) was used to extract clusters from keywords in the literature. A total of 28 clusters are generated. In order to better visualize, we only show the first 12 clustering categories in the timeline view (Figure 7B). The modular Q of all clusters was 0.935, with an average silhouette of 0.9841. This shows that it is a reliable, high-quality cluster graph diagram. From Figure 7B, it is obvious that the topics studied by these two clusters (#0 cortical activation, #1 pathological state) were recent research foci and may also become future research trends.

#### 3.2.4 Analysis of keywords with the strongest citation bursts

The “Burst words” represent keywords that are cited frequently over a period, thereby indicating the frontier areas. Figure 6B shows 15 burst keywords sorted by the “begin year.” As displayed, the related investigation started from the keyword “nitric oxide” from 2012 and lasted until 2017. And then, “laser acupuncture” became popular among researchers from 2013 to 2017. Currently, five keywords have become burst ones and last until now: quality, functional near-infrared spectroscopy, diagnosis, system and functional connectivity.

## 4 Discussion

### 4.1 General information

The annual number of articles showed a fluctuating rise with an increasing overall trend and was expected to grow in the future. On account of the progress of infrared imaging technology techniques and intensive research on the neural mechanism underlying acupuncture, it might indicate a promising direction in this field.

The largest quantities of publications are from China and the United States. Although the number of articles published in China is far greater than that of the United States, the average number of citations per article is less than half that of the United States. This result shows that the quality of Chinese articles has yet to be improved. Besides, the United States, Spain, and Germany were in the dominant positions according to centrality analysis. When it comes to research institutions, the most prolific was *Shanghai University of Traditional Chinese Medicine* while the top institutions with the highest centrality were Harvard University. The aforementioned phenomena suggested that despite China’s status as the birthplace of acupuncture, its abundance of TCM colleges and universities, numerous practitioners, and extensive publication output, it had yet to establish comprehensive collaborative relationships with institutions worldwide. Extensive collaboration helps researchers make the best use of resources and engage in a wider range of exchanges and discussions. It is essential for furthering the advancement of acupuncture research using infrared imaging technologies.

### 4.2 Research hotspots and global trend

The research hotspot and global trend of acupuncture infrared imaging technology were analyzed in terms of keywords and cited references. The literature with high co-citation typically exhibits superior quality and exerts significant influence within the research field, concurrently serving as a representation of the foundational knowledge in that particular domain. The significance of co-occurring keywords lies in their ability to encapsulate current research focal points and provide partial indications for future research directions.

#### 4.2.1 Acupoint and temperature

We analyzed the co-occurring keyword maps and found that “Temperature” and “Blood flow” ranked highly. A study on moxibustion found the increase in temperature led to an elevation in blood flow velocity due to the decrease in viscosity (Huang and Sheu, 2013). According to acupuncture theory, the therapeutic mechanism involves the stimulation of specific acupuncture points and meridians. Our study found that infrared imaging technology is often used to explore the physiological specificity and pathological changes of acupoints. Some studies believed that acupoint skin temperature may serve as a useful indicator of the health of nearby tissue and could even influence the choice of acupoints (Reddy et al., 2023). There were many studies using infrared technology to assess differences in acupoint skin temperature between patients and the general population, the temperature difference between the unaffected and affected sides or the temperature difference in the patient’s own treatment (Liu et al., 2020; Li et al., 2021; Reddy et al., 2023; Tu et al., 2023; Wang et al., 2023). An article published in 2012 shows asthma patients had significantly lower overall infrared radiation intensity at the left Taiyuan (LU 9) than that of healthy volunteers which suggested that IRT detection of acupoint temperature has a potential role in the diagnosis of diseases (Zhou et al., 2012). A systematic review suggested the possibility of thermally distinct between healthy and sick states at specific acupoints (Yang et al., 2022). In another study, the infrared thermal image was used to compare the temperature differences of the same acupoints on the bilateral sides to objectively assess the severity of Bell’s palsy (Liu et al., 2011). These findings hold significant implications for enhancing diagnostic accuracy, optimizing treatment point selection, and forecasting disease prognosis within clinical settings.

Nevertheless, due to the types of diseases studied and the limitations of research methods, there is no clear evidence that changes in skin temperature at specific acupoints predict pathological conditions. In the field of acupuncture infrared imaging technology, a large number of high-quality studies are still needed to explore the correlation between point temperature and pathological changes. Interestingly, in some studies, the specificity temperature of acupoint or meridians by infrared exploration is also seen as an exploration of traditional Chinese medicine (Yang et al., 2009; De Souza et al., 2016; Li et al., 2022).

#### 4.2.2 fNIRS and pain

In the co-occurring keyword maps, we found “Near infrared spectroscopy” and “pain” occupied an important position. Several systematic reviews have shown that acupuncture is a suitable treatment for relieving pain and improving quality of life (Manyanga et al., 2014). However, the neural mechanisms by which acupuncture alleviates pain have not been well understood. Pain can affect oxyhemodynamics in the prefrontal and sensory-motor cortices of the brain (Hall et al., 2021). Meanwhile, fNIRS can be used as a non-invasive neuroimaging method to provide long-term measures of cortical hemodynamics by measuring real-time changes of oxygenated (oxy-Hb) and deoxygenated hemoglobin (deoxy-Hb) (Peng et al., 2018). Moreover, fNIRS is superior to other brain imaging techniques such as fMRI in anti-motion interference. Recently, several studies have attempted to explore the therapeutic mechanisms of acupuncture analgesia using fNIRS. Yoshiki Morikawa’s study utilized near infrared spectroscopy (NIRS) and electrocardiography (ECG) to monitor prefrontal hemodynamic activity and autonomic activity. The results suggested that myofascial trigger points (MTrP) compression in the neck area modifies the activity of the autonomic nervous system via the prefrontal cortex to alleviate subjective pain (Morikawa et al., 2017). In the process of treating biliary colic with acupuncture, N. Sun and his team monitored brain neural activity with fNIRS, and correlation analysis will be conducted to investigate the relationship between pain relief and peripheral-cerebral functional changes (Sun et al., 2022).

However, its therapeutic mechanisms are still uncertain, partly because there is no method for providing a nonverbal objective assessment of pain. Therefore, some studies indicate that in the field of acupuncture, fNIRS has great potential for objective pain assessment in the clinical environment (Ding et al., 2016; Peng et al., 2018; Hall et al., 2021; Hu et al., 2021; Du et al., 2023). A systematic review and meta-analysis concluded Pain affects the prefrontal and sensory-motor cortices of the brain and can be measured by using fNIRS (Hall et al., 2021). Recent advance reported a complete suite of automated techniques for the computerized assessment of thermal images are employed to assess pain related thermal dysfunction (Herry and Frize, 2004). The objective assessment of pain by fNIRS helps to optimize the diagnosis of pain and the evaluation of the effectiveness of treatment, which is of great benefit to patients with specific pain, such as children, who are unable to self-report it. In addition, there are studies that suggest the use of fNIRS to predict the occurrence of pain (Hu et al., 2023).

#### 4.2.3 Acupuncture and functional connectivity

By analyzing the keyword bursts and the main content of the cited paper bursts, we have gained new insights into the future trends in this field. From 2017 to 2023, the main bursts keywords changed to functional near-infrared spectroscopy, quality, diagnosis, system and functional connectivity. The authors of the two strength burst references in recent years were both Raul Fernandez Rojas. Recent advances in fNIRS in acupuncture have focused on the effects of acupuncture interventions on cortical networks.

Functional connectivity refers to the statistical association or correlation between the activities of different brain regions. It reflects the degree of synchrony or coherence in neural activity between distinct brain regions. Functional connectivity analysis can provide valuable information on how the functional structure of the brain changes in response to stimulation. It promotes a deeper understanding of the mechanisms by which acupuncture improves brain function. In recent years, fNIRS has gained more attention by exploring the effects of acupuncture on brain regions and the function of functional connections between brain regions. A study observed the network connections with bilateral PFC as nodes showed significantly increased functional connectivity during acupuncture manipulation compared with controls through fNIRS (Si et al., 2021). Acupuncture is capable for increasing functional connectivity in dorsolateral PFC and primary somatosensory cortex thought fNIRS (Cao et al., 2023). A RCT including 70 patients employed the fNIRS to observe that, compared to patients taking antidepressants alone, depressed patients getting acupuncture in addition to antidepressants report improvements in their depression symptoms as well as greater resting state functional connectivity in the dorsolateral prefrontal cortex (DLPFC). Although it’s yet unknown if acupuncture lowers depression symptoms by altering DLPFC connectivity or if the altered connectivity is only a result of acupuncture’s therapeutic effect., it still offers an unbiased measurement to get beyond the issues with single-blinding in acupuncture-related depression research in the future (Wong et al., 2021).

PFC is the region of the cerebral cortex which covers the anterior portion of the frontal lobe. PFC receives input from all other cortical regions and functions to plan and direct motor, cognitive, affective, and social behavior across time. Research has indicated that acupuncture can be therapeutically applied to a range of neuropsychiatric conditions, such as neuropathic pains, Alzheimer’s disease, and Parkinson’s disease (Liang et al., 2014; Miranda et al., 2015; Jiang et al., 2018). Besides the clinical therapeutic effects, acupuncture is promising for enhancing human cognition and memory, and regulating emotion processing for healthy people (Hui et al., 2005; Zheng et al., 2018). However, the specific neural mechanism of acupuncture on the cerebral cortex remains unclear. Increasing evidence indicates that PFC’s adenosine triphosphate (ATP) level plays a role in the pathogenesis of depression. A study suggested that electroacupuncture (EA) can modulate extracellular ATP levels in the PFC of depressive-like MS rats, contributing to its antidepressant effects (Zheng et al., 2023). Another study using inflammatory bowel disease rat models showed that EA therapy has an alternative clinical use in the relief of visceral discomfort by lowering glutamine toxicity in the PFC (Jiang et al., 2023).

In this review, we discussed the application direction of infrared technology in the field of acupuncture, explored the relationship between acupoints and temperature, and provided potential help for the evaluation of clinical diagnosis and treatment effect of acupuncture. To explore the possibility of objective evaluation of pain with fNIRS and serve acupuncture clinic. In addition, our findings provide a new perspective on the use of fNIRS to explore the effects of acupuncture on functional connectivity between brain regions.

### 4.3 Applications of IRT in other fields and insights

In several studies in other fields, IRT is often used to explore the correlation between body surface temperature and pain. A retrospective study on persistent somatoform pain disorder (PSPD)compared the infrared thermograms of patients and healthy people. The results show the mean squared error and correlation values of the IR thermogram contribute to PSPD determination (Zou et al., 2023). The role of IRT as a complementary tool for the objective evaluation of pain is well established. Meanwhile, in the field of acupuncture, there have been several protocols that use IRT as an objective tool for assessing subjective pain in experiments (Hu et al., 2023). In the future, we expect IRT to be applied in more studies related to acupuncture analgesia to explore its potential as a complementary diagnostic and help us better explore the biological mechanisms of acupuncture.

## 5 Strengths and limitations

As far as we know, this study is the first to use literature measurement methods, visualization of the literature of 2008–2023 infrared imaging technology in the field of acupuncture, summarize the research status, and predict research trends. In addition, we utilized several software and methods to analyze the data. The conclusion was also interpreted from several angles. However, this study also has certain limitations. First, due to the software limitations of CiteSpace, our data come only from the WOS database. This may cause us to miss some highly cited literature. Besides, CiteSpace and VOSviewer are just visual literature analysis software. New trends and developments in terms of the analysis methods of infrared imaging technology, types of acupuncture, and diseases involved need to be further analyzed in the future.

## 6 Conclusion

This study demonstrates the various applications of infrared imaging in the acupuncture area. Due to its ability to visualize temperature in real time and to monitor blood flow in the brain, we firmly believe that infrared imaging technology brings tangible benefits to the field of acupuncture. We also expect that infrared imaging technology will advance and become more innovative, leading to its increased application in the field of acupuncture.

## Author contributions

YF: Writing – original draft. YX: Writing – original draft. BF: Writing – original draft. SL: Writing – original draft. ZZ: Writing – review & editing. JF: Writing – review & editing.

## Footnotes

^1^https://graphica.app/
